# Polydeoxyribonucleotide-delivering therapeutic hydrogel for diabetic wound healing

**DOI:** 10.1038/s41598-020-74004-0

**Published:** 2020-10-08

**Authors:** Da Yong Shin, Ji-Ung Park, Min-Ha Choi, Sukwha Kim, Hyoun-Ee Kim, Seol-Ha Jeong

**Affiliations:** 1 Department of Materials Science and Engineering, Seoul National University, Seoul, 08826 Republic of Korea; 2 Department of Plastic and Reconstructive Surgery, Seoul National University Boramae Medical Center, Seoul, 07061 Republic of Korea; 3 Medical Big Data Research Center, Seoul National University College of Medicine, Seoul, 03080 Republic of Korea; 4 Advanced Institutes of Convergence Technology, Seoul National University, Gwanggyo, Yeongtong-gu, Suwon-si, Gyeonggi-do 16229 Republic of Korea

**Keywords:** Health care, Materials science

## Abstract

Patients with diabetes experience delayed wound healing because of the uncontrolled glucose level in their bloodstream, which leads to impaired function of white blood cells, poor circulation, decreased production and repair of new blood vessels. Treatment using polydeoxyribonucleotide (PDRN), which is a DNA extracted from the sperm cells of salmon, has been introduced to accelerate the healing process of diabetic wounds. To accelerate the wound-healing process, sustained delivery of PDRN is critical. In this study, taking advantage of the non-invasive gelation property of alginate, PDRN was loaded inside the hydrogel (Alg-PDRN). The release behavior of PDRN was altered by controlling the crosslinking density of the Alg hydrogel. The amount of PDRN was the greatest inside the hydrogel with the highest crosslinking density because of the decreased diffusion. However, there was an optimal degree of crosslinking for the effective release of PDRN. In vitro studies using human dermal fibroblasts and diabetes mellitus fibroblasts and an in ovo chorioallantoic membrane assay confirmed that the Alg-PDRN hydrogel effectively induced cell proliferation and expression of angiogenic growth factors and promoted new blood vessel formation. Its effectiveness for accelerated diabetic wound healing was also confirmed in an in-vivo animal experiment using a diabetic mouse model.

## Introduction

As the aging population increases, we are facing numerous health-related challenges associated with chronic diseases. Diabetes is one of the most common diseases and is especially common in the elderly. A diabetic ulcer is a serious complication of diabetes that may lead to amputation and affects approximately 15% of patients diagnosed with diabetes^1,2^. The normal wound-healing process occurs through various cellular responses including the activation of keratinocytes, fibroblasts, endothelial cells, macrophages, and platelets^3^. However, diabetic wounds do not easily heal because of abnormalities in inflammatory cells, decreased or impaired growth factor formation^4^, new blood vessel formation^5^, collagen synthesis, and keratinocyte/fibroblast migration and proliferation^6–9^. Therefore, cell-based therapies have been adopted for treating unfavorable delayed healing conditions. Implantation of different cell types such as fibroblasts and keratinocytes in an absorbable collagen mesh has been performed^10–12^. This type of management is considered a positive treatment modality because the delivery of therapeutic biomolecules from implanted cells may result in successful angiogenesis for diabetic disorders^13^.

However, these autologous cell culture and transplantation techniques also have some disadvantages, such as the long period between the biopsy and grafting and the painful procedure for the patient^14,15^. Recently, an alternative diabetic-wound managing therapy using polydeoxyribonucleotide (PDRN) was introduced. PDRN is a DNA extracted from the sperm cells of Oncorhynchus *mykiss* (Salmon trout) or *Oncorhynchus keta* (Chum Salmon) with a molecular weight ranging from 50 to 1500 kDa^16^, and it is known to degrade by unspecific DNA nuclease then excreted by urine or feces. PDRN is known to have many positive therapeutic effects such as improving angiogenesis^6,17,18^, promoting osteoblast activity^19^, increasing collagen synthesis^20^ and having an anti-inflammatory effect^21^. Because of the ability of PDRN to promote the proliferation and migration of fibroblasts along with its effect on angiogenesis through the activation of A_2_A receptors and salvage pathways, it has been extensively studied in the field of diabetic wound-healing in pre-clinical studies^22^. Galeano et al. confirmed the effect of PDRN on diabetic defects through an in vivo study using a diabetic (db + /db +) mouse model. After 12 days of daily injection with PDRN in an incisional skin wound model of a db + /db + mouse, increased expression of vascular endothelial growth factor (VEGF) and CD31 was observed compared with that of the control group treated with NaCl in addition to increased wound breaking strength^23^. In a clinical case study, Kim et al. demonstrated that PDRN can improve peripheral tissue oxygenation and angiogenesis in diabetic ulcer patients and confirmed that tissue oxygenation was significantly improved at every time point (day 7, 14, and 28) with increased angiogenesis^24^. Despite the effect of PDRN on tissue repair, the application route was limited to intradermal or intramuscular injection in most of the studies. The need to inject PDRN places a burden on patients as many people suffer from needle phobia and visiting the hospital on a daily basis is inconvenient. In addition, the short half-life (~ 3 h) of PDRN makes daily injection inevitable^16^, resulting in painful procedures for the patients. Hydrogels are popular biomedical polymers that have been widely applied in the fields of drug delivery and soft tissue engineering. Hydrogels are composed of three-dimensional molecular networks that contain a large amount of water, making them excellent candidates for water-soluble drug delivery. They also exhibit high biocompatibility and provide a moist healing environment for accelerated regeneration of skin defects. These dressings can be made from either synthetic or natural polymers. Among the dressings available in hydrogel form, alginate (Alg) has a long history as an attractive dressing material owing to its excellent biocompatibility and intrinsic hemostatic property^25–27^. Alg is a polysaccharide composed of blocks of (1, 4)-linked β-D-manuronic acid (M) and α-L-guluronic acid (G) residues^28^. The ability to form a hydrogel through the interaction of the multivalent cations (e.g., Ca^2+^ or Ba^2+^) with the carboxyl groups in the sugar makes Alg a strong candidate as a drug-delivery vehicle. The mild gelation property of Alg minimizes any degenerative effects on the drug during the gelation process, and the loading efficiency of the drug can be maximized by mixing the drug in the precursor solution. The application of PDRN in diabetic tissue regeneration has already been proven to be beneficial, with a stimulatory effect on cell proliferation and new blood vessel formation. However, its application and delivery through a hydrogel vehicle has not yet been well defined.

In this work, we introduce a PDRN-loaded Alg hydrogel (Alg-PDRN), which could greatly reduce the burden of patients compared with the conventional injection method. The delivery and release behaviors of PDRN from Alg hydrogel with various alginate concentrations were evaluated along with its therapeutic effects. Characterization of PDRN and the crosslinking density of the hydrogel was performed, and the rheological behavior, gelation time, swelling ratio, and PDRN release behavior were also monitored. In addition, the in vitro cellular responses were evaluated using normal human dermal fibroblasts (HDFs), diabetes mellitus (DM) fibroblasts, and human umbilical vein endothelial cells (HUVECs). In addition, an in ovo chorioallantoic membrane (CAM) assay was performed to evaluate the angiogenic effect of the PDRN-loaded hydrogel. Finally, in vivo analysis of the PDRN-loaded hydrogel carrier was performed in a full-thickness wound model on a diabetic mouse, and the results were compared with those of the PDRN-injected wounds.

## Results and discussion

### Internal gelation of Alg-PDRN

Alg-PDRN was prepared through the internal gelation of Alg. Briefly, a mixture of CaCO_3_ and various concentrations of Alg was prepared together with PDRN (100 µg/ml), and the addition of D-glucono-δ-lactone (GDL) initiated the release of Ca^2+^ ions. The released Ca^2+^ ions cross-linked Alg by forming egg-box junctions.

Table S1 shows the amount of each reagent added to produce the 2%Alg-PDRN, 3%Alg-PDRN, 4%Alg-PDRN, and 5%Alg-PDRN. A ratio of 1 w/v% Alg:12.5 mM CaCO_3_ was maintained, and the amount of GDL added increased with increasing CaCO_3_ content with a fixed molar ratio of 0.5. The slowly dissolving CaCO_3_ enabled homogeneous gelation, which is suitable for sheet-type hydrogel formation, unlike the conventional external gelation methods involving CaCl_2_ as the Ca source^29^. In many previous papers, the degree of crosslinking of Alg hydrogel prepared through internal gelation using CaCO_3_ and GDL was altered by varying the concentration of CaCO_3_ with a fixed volume of Alg^30,31^. However, in this work, instead of varying the concentration of CaCO_3_ to control the crosslinking density, the ratio of Alg to CaCO_3_ was fixed and the concentration of Alg solution was varied to achieve different degrees of crosslinking. Because Alg itself is known to have a stimulatory effect in wound healing^25,32^, increasing the Alg content in the hydrogel was intended to increase the biocompatibility of the hydrogel as well as the crosslinking density.

### Characterization of Alg-PDRN and PDRN

**Figure 1:**
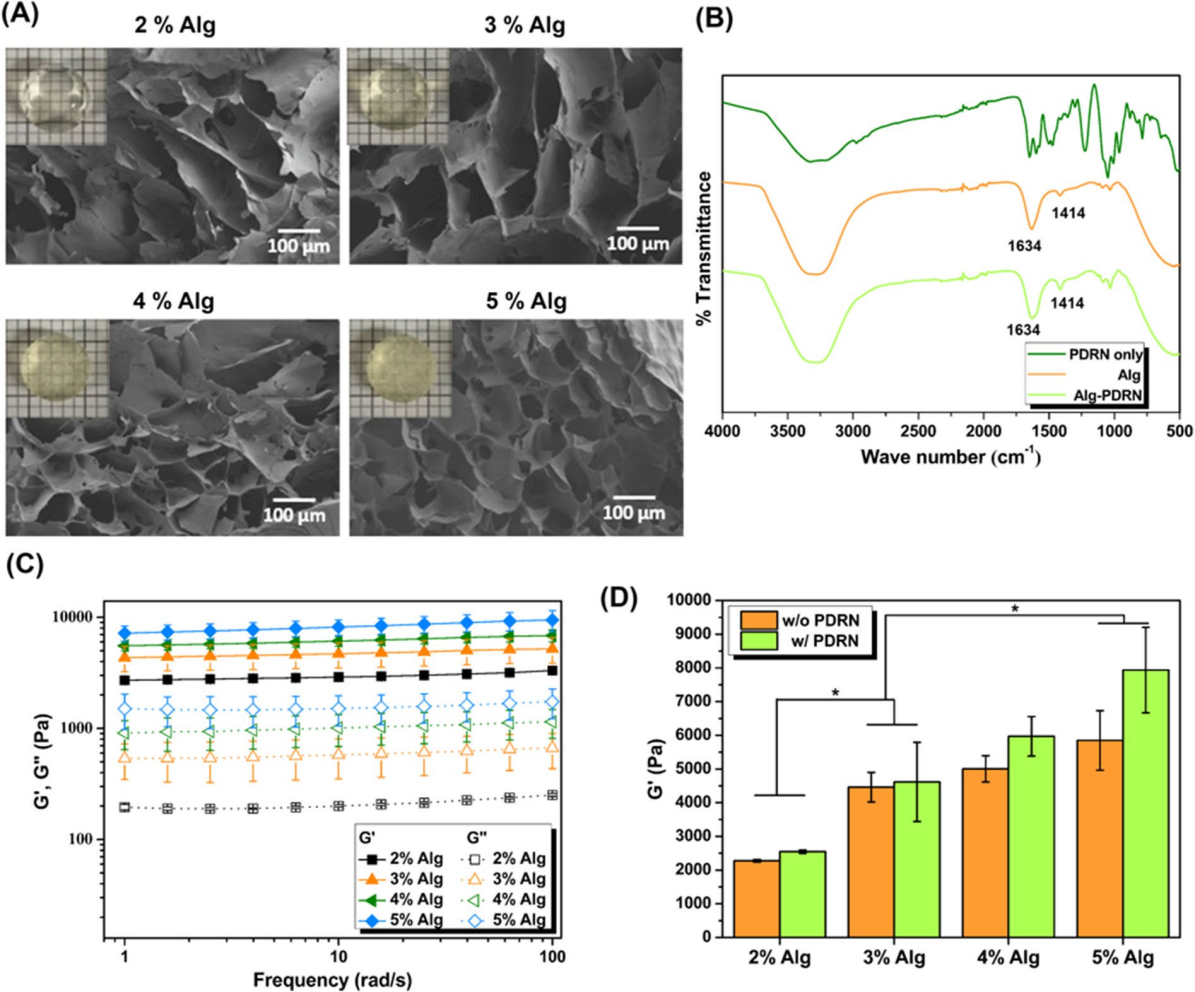
(**A**) Cross-sectional SEM images of Alg-PDRN with differet Alg concentrations (inset: optical images of hydrogels). (**B**) FT-IR spectra of PDRN only, Alg, and Alg-PDRN hydrogel. (**C**) Rheological behavior of Alg-PDRN under frequency sweep mode. (**D**) Storage modulus of Alg hydrogels with and without PDRN at 1 Hz (*p < 0.05).

Optical images and low-magnification cross-sectional SEM images of the Alg-PDRN are presented in Fig. 1A.

The transparency of Alg-PDRN decreased with increasing Alg concentration, and the pore size of the hydrogels also showed a decreasing tendency with increasing Alg concentration. These results indicate that the degree of crosslinking increased with increasing Alg concentration^33^. The swelling ratio and gelation time were measured, and the results are presented in Table 1. The swelling ratio decreased from approximately 50 to 25 g/g as the Alg concentration increased from 2 to 5%. The gelation time also decreased with increasing Alg solution, indicating that gelation occurred faster with a higher Alg concentration.

**Table 1:** Equilibrium swelling ratio and gelation time of Alg-PDRN as a function of Alg concentration.

Sample	Equilibrium swelling ratio [g/g]	Gelation time [min]
2% Alg-PDRN	49.19 ± 0.84	51.55 ± 6.60
3% Alg-PDRN	37.42 ± 0.66	31.25 ± 2.38
4% Alg-PDRN	28.98 ± 0.31	22.40 ± 1.26
5% Alg-PDRN	25.32 ± 0.37	19.76 ± 0.22

FT-IR spectra of PDRN only, Alg, and Alg-PDRN are presented in Fig. 1B. Characteristic Alg peaks were observed at 1634 cm^−1^ (C=O bond) and 1414 cm^−1^^34^. The characteristic peaks of Alg hydrogel and Alg-PDRN hydrogel were identical, indicating that no specific bond formation occurred between the Alg polymer chains and PDRN.

### Rheological behavior

The rheological behavior of Alg-PDRN was explored using a strain-controlled rheometer, as shown in Fig. 1C. The storage modulus (G′) values of all the Alg-PDRN were greater than the loss modulus (G″) values under frequency sweep mode, indicating that they behaved like solid gels with well-cross-linked polymer chains. G′ of the hydrogel increased with increasing Alg concentration. At 1 Hz, G′ increased from approximately 2500 Pa for 2% Alg to 7900 Pa for 5% Alg. Thus, the degree of crosslinking increased with increasing Alg concentration. Figure 1D compares the storage moduli of Alg and Alg-PDRN with different Alg concentrations at 1 Hz, the representative frequency used for strain sweep experiments to determine the linear viscoelastic region. At low Alg concentration, no significant differences in G′ of the Alg and Alg-PDRN hydrogels were observed. However, at high Alg concentration, G′ of Alg-PDRN was greater than that of Alg. Upon increasing the Alg concentration from 4 to 5%, the storage modulus of Alg-PDRN increased from approximately 5900 to 7900 Pa, whereas that of Alg increased from approximately 5000 to 5800 Pa, respectively. The difference between the two groups became significant only at high Alg concentration.

The higher Alg concentration resulted in a higher crosslinking density, which enabled a higher degree of physical entanglement between PDRN and the Alg polymer chain. This trend is observed in the rheological behavior in Fig. 1D, where the storage modulus of Alg-PDRN sharply increased compared with that of Alg at Alg concentrations of 4% and 5%. This phenomenon can be explained by the strengthening of the hydrogel by the presence of PDRN, which acted as a semi-interpenetrating polymer network^35^. The homogeneous dispersion of PDRN led to an increased stress transfer from polymer chains to PDRN, similar to the effect of nano fillers such as carbon nanotubes, cellulose nano crystals, and nano clay^36^. Although the strength of PDRN itself is very low compared with strengthening fillers such as carbon nanotubes or nano crystals, the high degree of physical entanglement of the two polymer materials at high Alg concentration was sufficient to slightly increase the strength of the hydrogel. The similar storage moduli of Alg and Alg-PDRN at Alg concentrations of 2% and 3% resulted from the lack of interaction between the alginate polymer network and PDRN as they have a relatively larger mesh size, enabling the free floating of PDRN.

**Figure 2:**
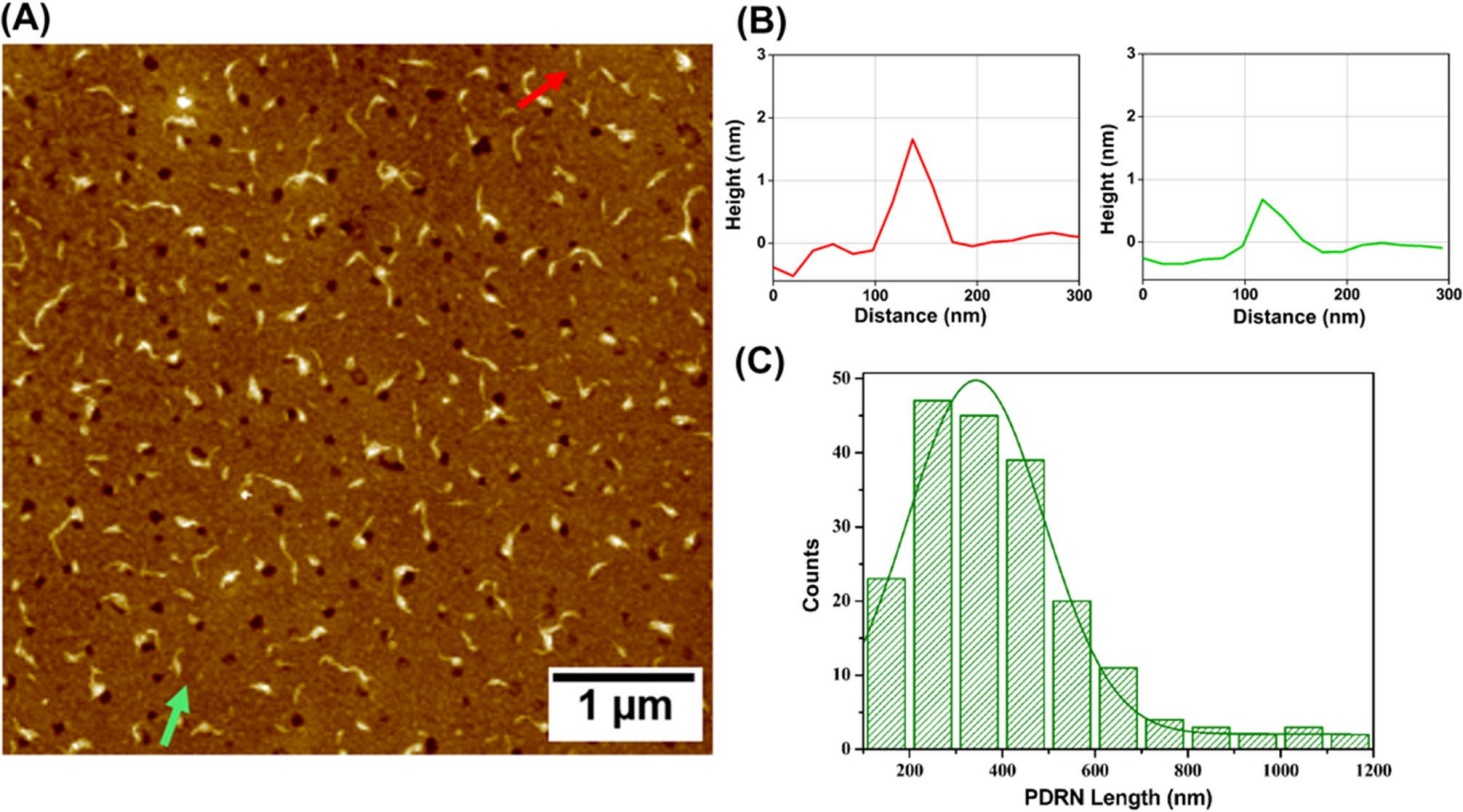
(**A**) AFM image showing PDRN morphology. (**B**) Line profile of the height of PDRN indicated by the red and green arrows in (**A**). (**C**) Length distribution of PDRN.

The long and thin thread-like morphology of PDRN is observed in the atomic force microscopy (AFM) images in Fig. 2A.

The thickness of PDRN varied from approximately 0.5 to 2.5 nm as the thread-like PDRN also existed in bundles (Fig. 2B). The length of PDRN ranged from approximately 100 to 1200 nm with an average length of approximately 400 nm, as observed in Fig. 2C. The incorporation of this thin thread-like PDRN with varying length into the Alg hydrogel with different crosslinking density resulted in different release behaviors of PDRN from the hydrogel carrier in addition to different rheological behaviors.

### PDRN Release Behavior

**Figure 3:**
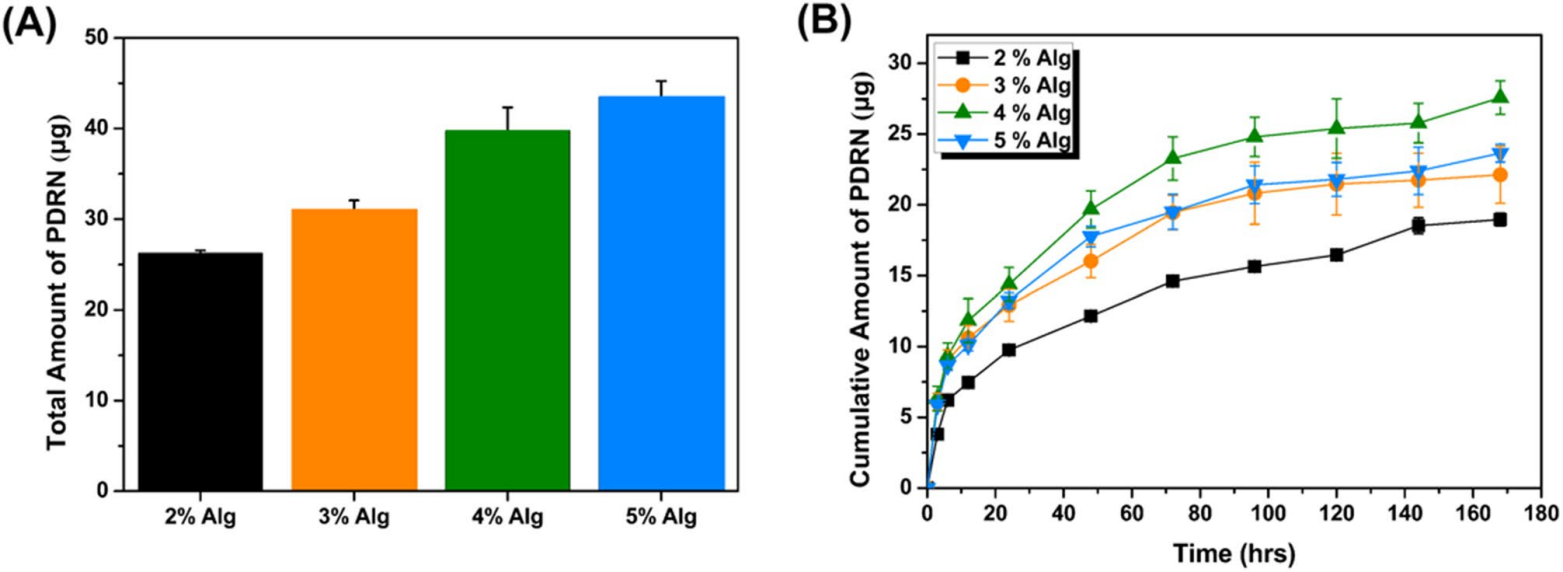
(**A**) Total amount of PDRN remaining inside the Alg-PDRN hydrogel carrier after surface extraction and (**B**) PDRN releasing profile of the Alg-PDRN hydrogels with different Alg concentration.

The amount of PDRN remaining after the initial release and the amount of PDRN released from the hydrogel to the PBS solution over 7 days are shown in Fig. 3. The amount of PDRN that diffused into the PBS solution under the membrane was quantified using UV–vis spectroscopy by observing the absorbance at a wavelength of 260 nm^37^. A standard curve was obtained by measuring a determined amount of PDRN dissolved in PBS, as shown in Fig. S2. A representative curve of PDRN absorbance released from the hydrogel was also recorded, with an absorbance peak appearing at 260 nm. The total amount of PDRN remaining after surface extraction, which is a technique often used to control the initial burst release of a drug in drug delivery using hydrogel carriers, to avoid the burst release effect, increased with increasing Alg concentration, as shown in Fig. 3A. The high crosslinking density of the hydrogels with higher Alg concentration minimized the loss of PDRN during the surface extraction process. Although the greatest amount of PDRN remained in 5%Alg-PDRN, the greatest amount of PDRN was released from 4%Alg-PDRN over the 7-day period, as shown in Fig. 3B.

Figure S3 represents the releasing behavior of PDRN in cumulative percent release. Because the initial loading amount of PDRN was different among the samples with different Alg concentration after surface extraction, the percent cumulative release of PDRN shows a completely different trend compared to cumulative amount of PDRN released. This difference is due to the different loading amount of PDRN after surface extraction and the releasing apparatus that only exposes one surface of the hydrogel to the releasing medium. Because smallest amount of PDRN was loaded in the center of the hydrogel for 2% Alg-PDRN, the initial releasing percentage was not very rapid. However, due to the relatively big pore sizes, after certain amount time, rapid release of the remaining PDRN occurred. On the other hand, Alg-PDRN with higher concentration had more PDRN remaining in the center of the hydrogel (close to the surface of the hydrogel) therefore, showing a rapid percentage release during the initial stage and later on a slower release due to the smaller pore size. 5% Alg-PDRN showed the slowest percent release among sample due to its small pore size which was coherent with the cumulative amount release data.

The degree of physical entanglement between PDRN and Alg directly affected the release behavior and loading efficiency of PDRN from the hydrogel carrier. The higher degree of crosslinking in the high-Alg-concentration hydrogel minimized the loss of PDRN during the surface extraction procedure, whereas more PDRN was washed away during that process in the lower-Alg-concentration hydrogels because of their larger mesh size. The small thickness of PDRN (approximately 0.5–2.5 nm) enabled their release through the meshes of the hydrogel; however, physical entanglement resulting from their relatively long lengths (100–1200 nm) resulted in stable encapsulation of PDRN inside the hydrogel with increasing Alg concentration. This phenomenon is well illustrated by the release behavior of PDRN, where less PDRN was released from 5%Alg-PDRN than 4%Alg-PDRN. The high degree of physical entanglement between PDRN and the polymer chain is indirectly demonstrated by the sharp increase in the storage modulus of 5%Alg-PDRN compared with that of 5%Alg; the difference in storage modulus between Alg and Alg-PDRN was the greatest at the 5% Alg concentration. Although the greatest amount of PDRN remained after surface extraction, the release behavior of PDRN from 5% Alg-PDRN was retarded compared with that from 4%Alg-PDRN because of the greater interaction between the two polymers. Therefore, of the materials examined in our study, we concluded that 4%Alg-PDRN was the optimal composition for delivering PDRN to the wound bed with high loading efficiency and constant release behavior.

### In vitro cell tests

**Figure 4:**
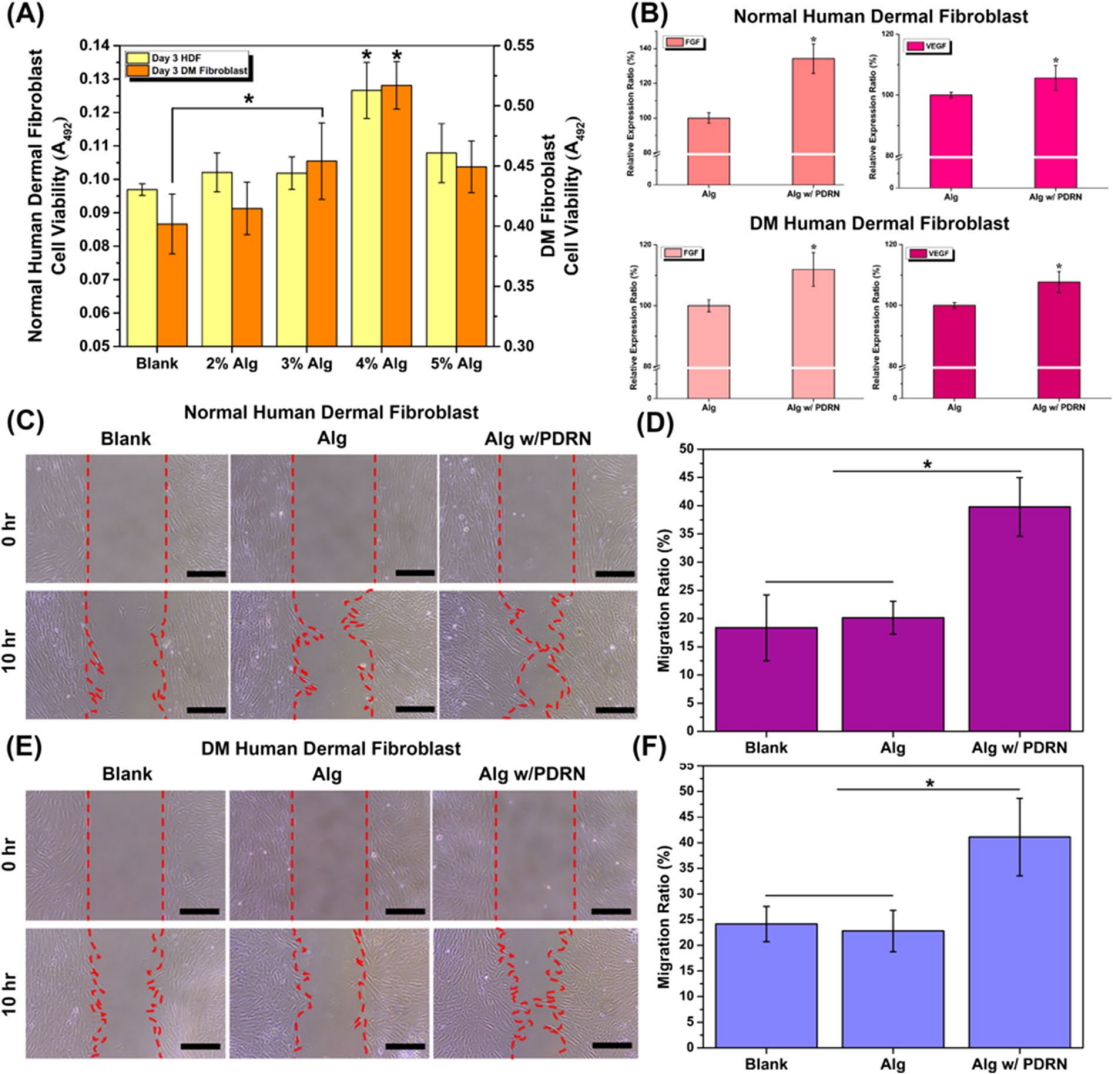
(**A**) Cell viability of normal HDF and DM fibroblasts treated with Alg-PDRN for 3 days. (**B**) FGF and VEGF expression from normal HDF and DM fibroblasts treated with Alg and Alg-PDRN. (**C**) Cell migration images of normal HDF and (**D**) migration ratio of blank, Alg and Alg-PDRN (scale bar = 250 µm). (**E**) Cell migration images of DM fibroblasts and (**F**) migration ratio of blank, Alg and Alg-PDRN (*p < 0.05).

The activity of fibroblasts is known to be suppressed in diabetic patients, therefore, it is meaningful to compare the effect of PDRN on normal HDF and fibroblast from diabetic patients. In order to indirectly evaluate the effect of the PDRN released from the hydrogel carrier on diabetic wound healing, the cell proliferation, migration, and enzyme-linked immunosorbent assays were performed on both normal human dermal fibroblasts (HDFs) and diabetes mellitus (DM) fibroblasts as illustrated in Fig. 4.

The viability of the cells after 3 days of culturing was evaluated using an MTS assay since dressing materials are usually changed every 3 or 4 days, and the results are presented in Fig. 4A. After 3 days of culturing, the cell viability of both the normal and DM fibroblasts were higher for Alg-PDRN than for the blank. Although the differences were not all statistically significant, the cell viability showed an increasing tendency with increasing Alg concentration until 4% Alg-PDRN and then slightly decreased at 5% Alg-PDRN, which is consistent with the release behavior data. The 4% Alg-PDRN treated group, which released the greatest amount of PDRN, exhibited significantly higher cell viability than all the other groups for both cell types, indicating that a large amount of continuously released PDRN is beneficial for the cell proliferation of both normal and diabetic fibroblasts. In diabetic wounds, the regeneration of blood vessels is usually impaired; therefore, enhancing angiogenesis is a critical factor that could improve the healing process^38^. To determine whether the released PDRN could lead to enhanced angiogenesis, the quantities of the angiogenic factors, including fibroblast growth factor (FGF) and VEGF, expressed from normal and DM fibroblasts were measured, as shown in Fig. 4B. The quantities of both FGF and VEGF were significantly higher in the Alg-PDRN-treated group than in the control group without PDRN, indicating that continuously released PDRN could stimulate the expression of FGF and VEGF, which will contribute to enhanced angiogenesis.

To evaluate the effect of the released PDRN on cell migration, scratch tests were performed. Stimulation of cell migration is known to play a crucial role in wound repair.^39^ An in vitro scratch assay was used to evaluate the effect of Alg-PDRN on normal and diabetic fibroblast migration, as shown in Fig. 4C–F. Figure 4C and E present the results of the cell migration assay after 10-h incubation, where the blank is the control group with no hydrogel, Alg is the 4%Alg hydrogel without PDRN, and Alg-PDRN is the 4% Alg hydrogel with PDRN, which showed the highest proliferation in the MTS assay. The Alg-PDRN significantly stimulated the migration of both normal and diabetic fibroblasts compared with Alg or the control group, as observed in Fig. 4D and F, indicating that the released PDRN successfully stimulated the migration of fibroblasts.

Finally, the effect of the Alg-PDRN hydrogel on HUVECs was evaluated through cell proliferation and enzyme-linked immunosorbent assays, as shown in Fig. S4. The viability of HUVEC was evaluated using an MTS assay after 3 days of culturing, as shown in Fig. S4A. After 3 days of culturing, the viability of HUVECs for Alg-PDRN was higher than that of the blank without the hydrogel, and the trend was similar to that of the fibroblast cells: increasing cell viability with increasing Alg concentration up to 4% followed by a slight decrease at 5% Alg concentration. Notably, the group treated with 4% Alg-PDRN exhibited a significantly higher cell viability compared with all the other groups, and comparison of the proliferation of the fibroblasts indicated that the release of PDRN from the hydrogels was more effective on HUVECs. The expression of VEGF from HUVECs was also measured, as shown in Fig. S4B. The expression level of VEGF was significantly higher in the Alg-PDRN-hydrogel-treated group than in the blank-Alg-hydrogel-treated group.

The viability of both types of fibroblasts and HUVECs increased with increasing Alg concentration up to 4% and slightly decreased at 5%, which is related to the released amount of PDRN. Because the greatest amount of PDRN was released from the 4% Alg-PDRN, it successfully delivered a sufficient amount of PDRN continuously to stimulate the cells. Many previous studies have confirmed that PDRN can stimulate the expression of VEGF, synthesis of collagen, and proliferation and migration of fibroblasts for accelerated granulation tissue formation^40,41^.

### In-ovo CAM assay

**Figure 5:**
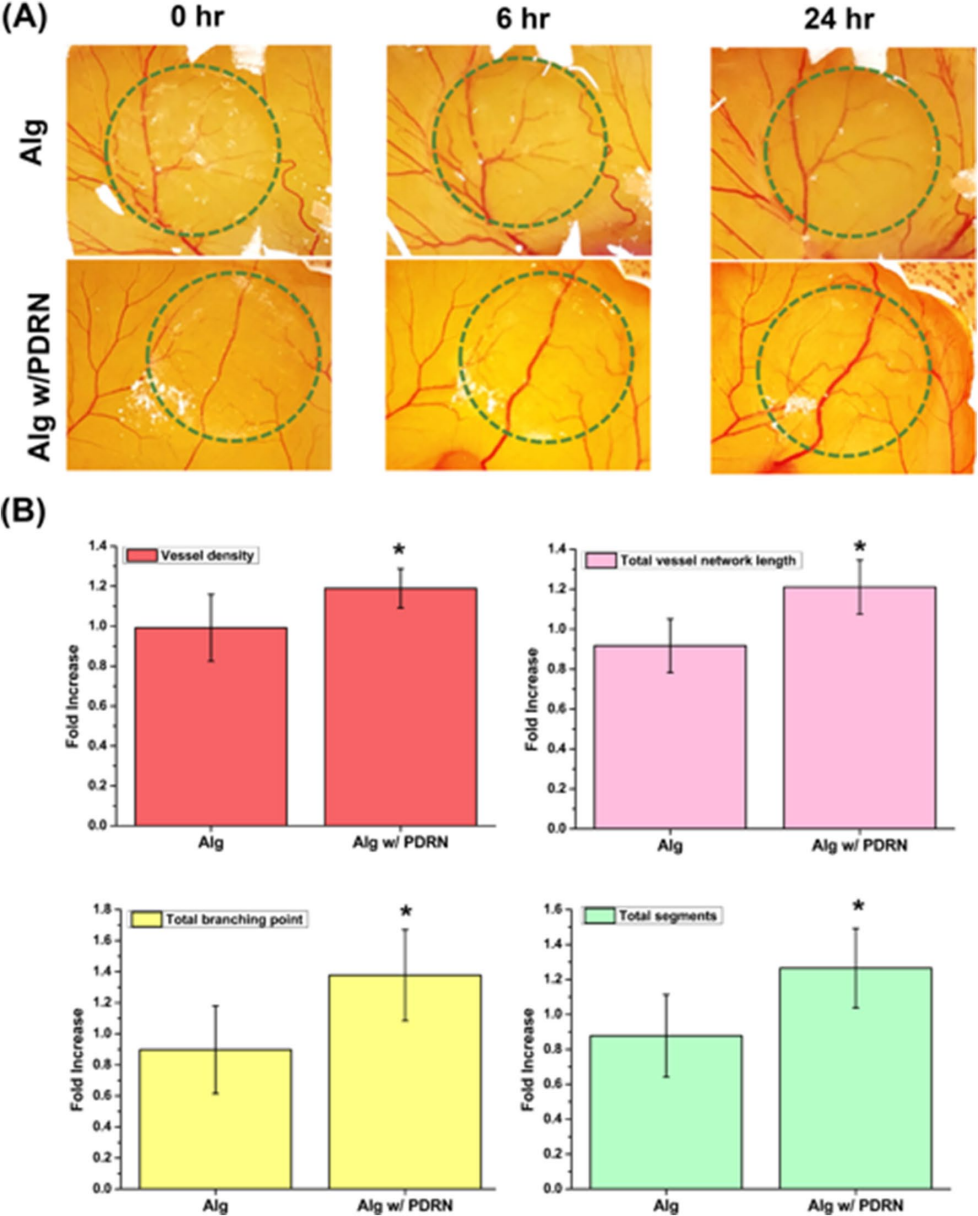
(**A**) Representative CAM images with Alg and Alg-PDRN at 0, 6 and 24 h. (**B**) Quantitative analytical results of vessel density, total vessel segment length, total branching points and total segments at 6 h (*p < 0.05).

The angiogenic effect of Alg-PDRN was evaluated using a CAM assay, as shown in Fig. 5. Alg and Alg-PDRN were placed on a chorioallantoic membrane, as shown in Fig. 5A, and the vessel formation was monitored for 6 and 24 h. The Alg-PDRN-treated group exhibited increased vessel formation. The quantitative analysis in Fig. 5B also reveals that the Alg-PDRN-treated group exhibited higher vessel density and total vessel network length, with an increased number of branching points and total segments at 6 h. At 24 h, the differences were not significant because the fast development of the embryo resulted in rapid new vessel formation. The stimulatory effect of PDRN released from the hydrogel was best represented at 6 h.

The in vitro results of the current study are consistent with previous findings, suggesting that Alg-PDRN can stimulate the biological responses needed for diabetic wound regeneration in the same manner as PDRN injection; the elimination of the burdensome injection while achieving the same enhanced biological responses is the key finding of this research.

### In vivo animal experiment

**Figure 6:**
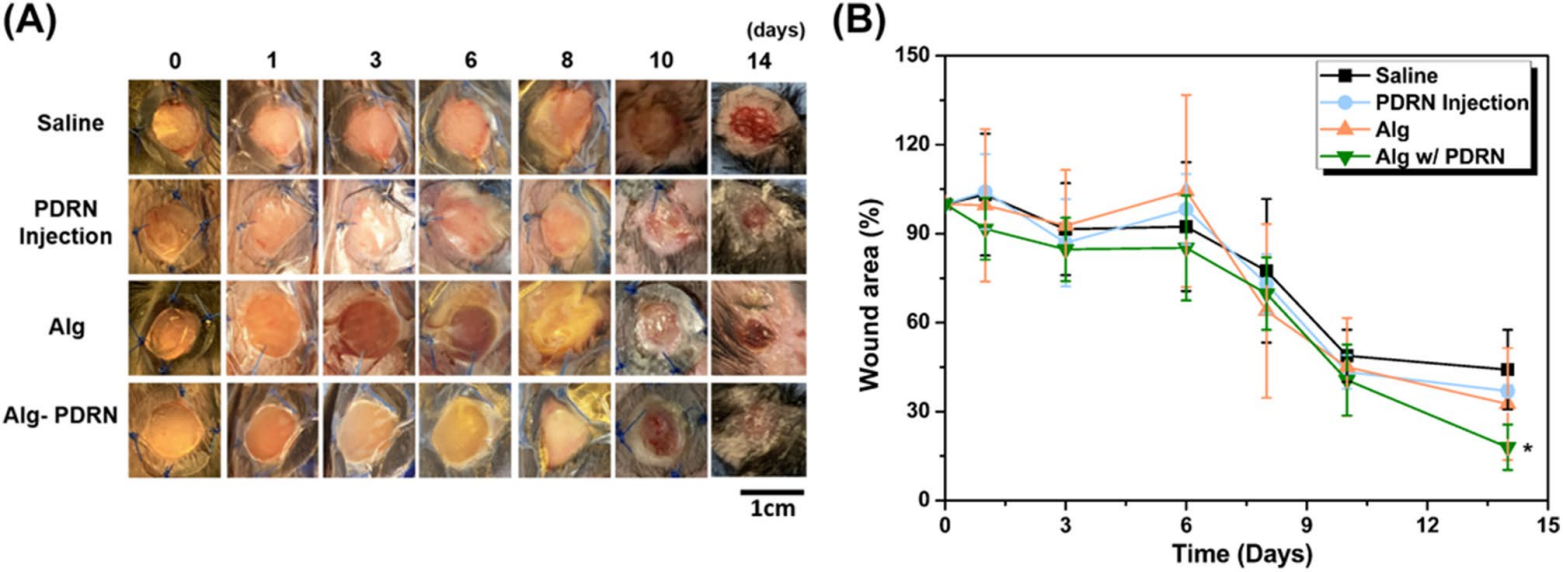
Healing rate of saline, PDRN injection, Alg and Alg-PDRN treated wound: (**A**) macroscopic appearance of the excised wound at different time points and (**B**) the remaining wound area after 1, 3, 6, 8, 10 and 14 days of treatment (n = 5, *p < 0.05).

The in-vivo assessment was performed by comparing the groups treated with saline injection (saline), PDRN injection, Alg hydrogel, and 4%Alg-PDRN hydrogel, which exhibited the best performance in vitro. The healing rate of the excised wound of a db + /db + mouse was monitored for 14 days and expressed as remaining wound area (%) to quantitatively compare the regenerated part with its original excised size, as shown in Fig. 6A.

There were no significant differences in defect size among the groups up to day 10; however, at day 14, complete re-epithelialization was observed for the groups treated with the hydrogels but not for the saline-or PDRN-injected groups. Quantitative analysis of the wound area also revealed similar trends as the macroscopic images, with the group treated with Alg-PDRN exhibiting a significantly faster wound closure rate than the saline-treated group. The remaining wound area at day 14 was approximately 44%, 37%, 32%, and 18% for the saline-injected, PDRN-injected, Alg-treated, and Alg-PDRN-treated groups, respectively. Initial expansion of the wound was observed at day 6 (Fig. 6B). This expansion is often observed in an excisional wound model during the early stage of the healing process^42^ because of loosening of the tissue; however, it did not affect the whole regeneration process. The Alg-PDRN-treated group appeared to exhibit the fastest healing rate; however, the dramatic effectiveness of the continuously released PDRN was masked by the effectiveness of the Alg hydrogel itself as Alg is well known for its ability to promote re-epithelialization^43,44^. Therefore, further histochemical analysis was performed to thoroughly examine the tissue regeneration of the db + /db + mouse treated with Alg-PDRN.

**Figure 7:**
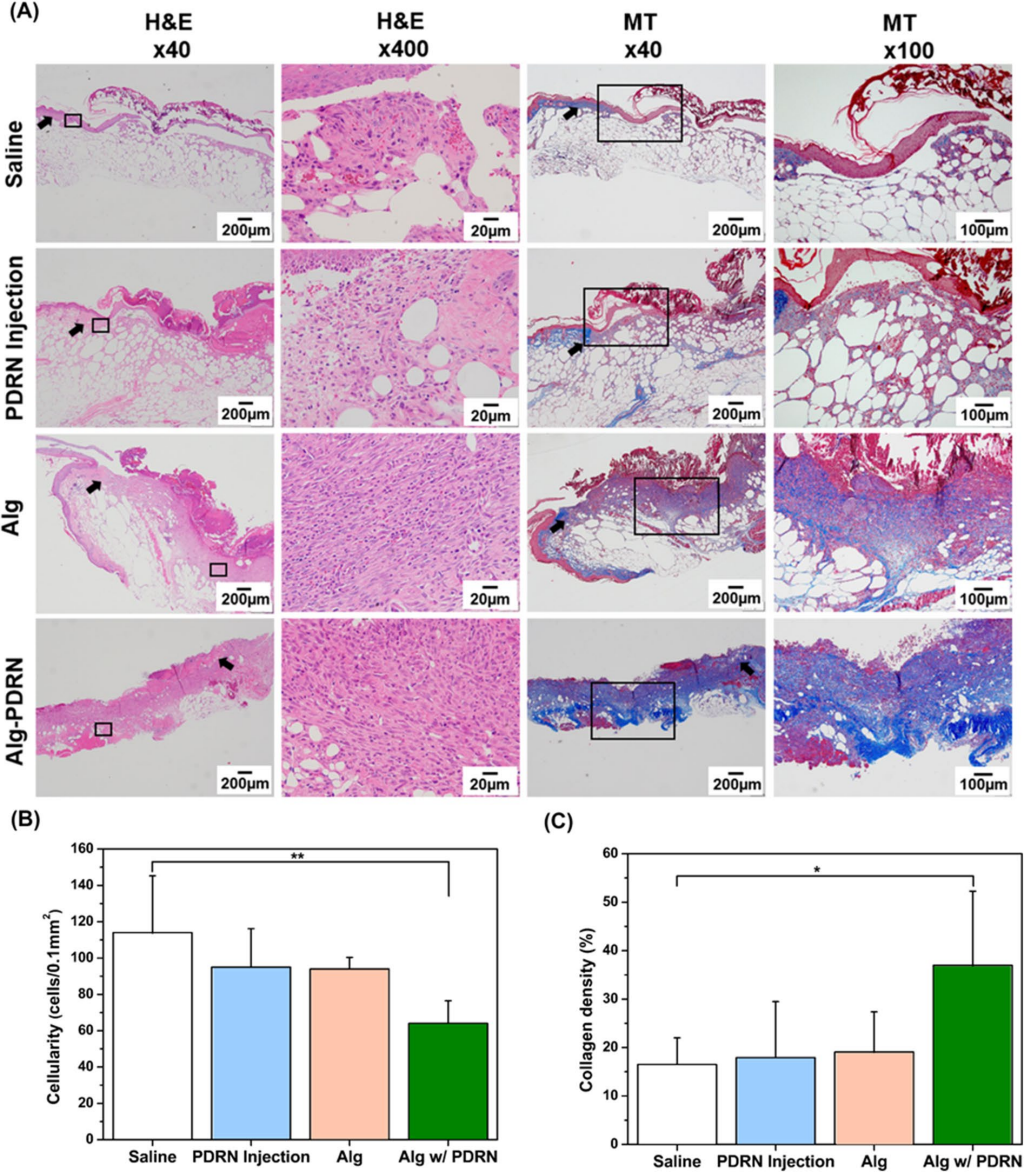
Histological examination of the wound region after 14 days of treatment: (**A**) representative H&E staining (Original magnification: X40—Scale bar = 200 µm, original magnification: X400—Scale bar = 20 µm) and MT staining images (Original magnification: X40—Scale bar = 200 µm, original magnification: X100—Scale bar = 100 µm) of the wound region and (**B**) quantitative analysis of cellularity and collagen density (*p < 0.05, **p < 0.005) (MT: blue—collagen, red—keratin/muscle fiber) (arrow: wound edge).

Histological analysis of H&E and MT stained tissue was performed, as shown in Fig. 7A.

It is apparent that regeneration did not occur for the saline-injected group. However, partial re-epithelialization and almost completed re-epithelialization was observed for the PDRN-injected group and both hydrogel-treated groups, respectively. The thickness of the granulation tissue appeared to be greater for the hydrogel-treated groups than for the other two groups, confirming that the Alg hydrogel promoted the regeneration of the wounds. This finding was consistent with the wound closure data. Cellularity assessment was performed and quantified from the H&E stained images (Fig. 7B). The number of inflammatory cells decreased in the order of saline-injected > PDRN-injected > Alg-treated > Alg-PDRN-treated with 114, 95, 94, and 64 cell/0.1 mm^2^, respectively. Although the PDRN-injected and Alg-treated groups did not show any statistical difference in terms of the number of inflammatory cells compared with the saline-injected group, the Alg-PDRN-treated group contained a significantly decreased number of inflammatory cells compared with the saline-injected group. The inflammatory stage is a phase in the wound-healing process, and the number of inflammatory cells are known to decrease as the wound recovers^45^. Therefore, it can be concluded that the wound-healing process further progressed for the group treated with Alg-PDRN owing to the effect of the continuously released PDRN. The regenerated collagen density was also determined from the MT stained images (Fig. 7C). The collagen density of the regenerated tissue was approximately 16%, 18%, 19%, and 37% for the saline-injected, PDRN-injected, Alg-treated, and Alg-PDRN-treated groups, respectively. As reported in previous studies, the low density of collagen in the regenerated tissue decreases the elasticity of the repaired region, resulting in mechanical mismatch between the new and original skin tissue^46^. The significantly higher regenerated collagen density of the Alg-PDRN-treated group compared with that of the saline-treated group is especially meaningful because the collagen synthesis is usually impaired in diabetic patients, resulting in delayed wound healing^47^. It can be concluded that the continuous release of PDRN from the Alg hydrogel carrier can successfully promote the deposition of collagen in diabetic wounds.

**Figure 8:**
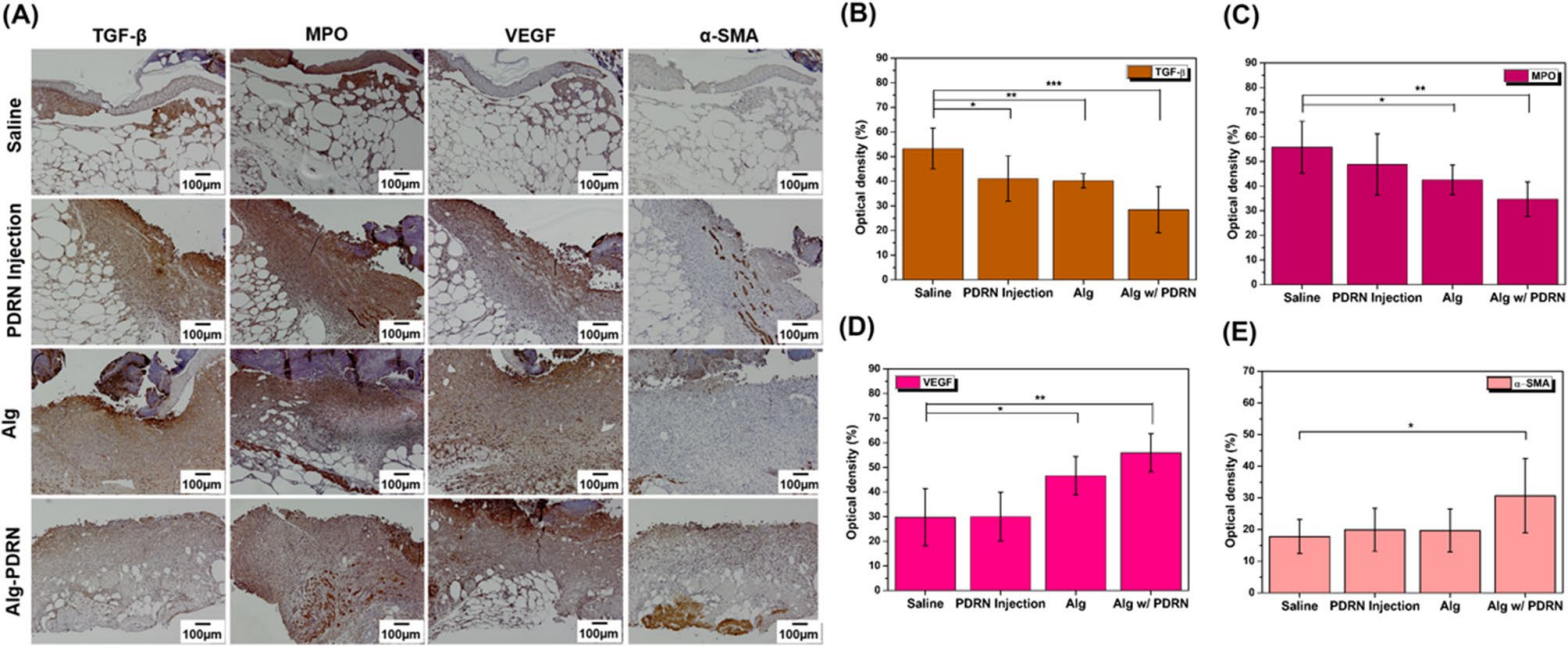
Immunohistochemistry of the wound region after 14 days of treatment: (**A**) representative images of TGF-β, MPO, VEGF and α-SMA stained images of the wound region and quantitative analysis of (**B**) TGF-β, (C) MPO, (D) VEGF and (E) α-SMA (scale bar = 100 µm, *p < 0.05, **p < 0.005, ***p < 0.0005).

Finally, immunohistochemistry assessment of TGF-β, MPO, VEGF, and α-SMA was performed, as shown in Fig. 8A. Both TGF-β (Fig. 8B) and MPO (Fig. 8C) showed a decreasing trend from approximately 53% to 28% and from approximately 55% to 35% for the saline-injected group and Alg-PDRN-treated group, respectively. Both markers, which are closely related to inflammatory responses^48,49^, were suppressed for the Alg-PDRN-treated group because the recovery of the wound had proceeded further than for the other groups, as indicated by the cellularity data in Fig. 8B.

Impaired blood vessel formation is one of the main reasons for impaired wound healing in diabetic wounds along with decreased collagen synthesis. The VEGF expression from the group treated with Alg-PDRN was significantly higher (approximately 56%) than that of the saline-injected group (approximately 30%) (Fig. 8D). However, that of the PDRN-injected group (approximately 30%) did not show any statistical difference with that of the saline-injected group. This finding indicates that PDRN, originally known to promote angiogenesis, is more effective when released continuously to the wound bed than when injected once because of its low half-life. The expression of α-SMA was also significantly higher in the Alg-PDRN-treated group than in the saline-injected group (Fig. 8E); this finding is consistent with the collagen density data as α-SMA is a marker of myofibroblast responsible for collagen production^50^. Thus, the in-vivo assessment confirmed the benefit of the Alg-PDRN hydrogel treatment for diabetic wound regeneration over the PDRN injection method.

## Conclusions

In summary, we discussed the potential of using Alg-PDRN hydrogel with homogeneous microstructure for enhanced wound healing in diabetic patients. The degree of crosslinking was controlled by adjusting the Alg concentration to control the release behavior of PDRN from the hydrogel carrier, and the 4%Alg-PDRN hydrogel was the optimum carrier for efficient PDRN delivery in this study. In vitro experiments confirmed a higher expression of angiogenic growth factors along with cellular proliferation. The stimulatory effect of the 4%Alg-PDRN hydrogel on blood-vessel formation was also confirmed through an in ovo CAM assay. In vivo studies also demonstrated that the Alg-PDRN hydrogel could be applied to efficiently repair diabetic skin wounds to a comparable degree as the conventional PDRN-injection method, thereby reducing the inconvenience to patients. These results suggest that incorporating PDRN within a hydrogel carrier can be used to achieve sustained delivery of the DNA with stimulatory biological effects while eliminating the use of needles, indicating the great potential for its use in non-invasive diabetic wound healing.

## Materials and Methods

### Materials and preparation of PDRN-loaded alginate hydrogel

Sodium alginate (alginic acid sodium salt; Sigma-Aldrich, USA) in salt form, calcium carbonate (CaCO_3_, Sigma-Aldrich, USA), ammonium fluoride (NH_4_F, Sigma-Aldrich, USA), D-( +)-gluconic acid δ-lactone (GDL; Sigma-Aldrich, USA), and PDRN (PDRN; Genoss, South Korea) were prepared to fabricate the Alg-PDRN hydrogel according to literature^51^. Briefly, mixture of 2–5 w/v% Alg with a calculated amount of CaCO_3_ was prepared in distilled water (DW) under continuous stirring with a fixed Alg to calcium carbonate ratio of 1 w/v%:12.5 mM, as shown in Table 1. The amount of GDL was set to achieve a CaCO_3_ to GDL molar ratio of 0.5 to attain a neutral pH^51^. The samples were named according to the Alg concentration as 2%Alg-PDRN, 3%Alg-PDRN, 4%Alg-PDRN, and 5%Alg-PDRN; PDRN was loaded in each sample at a concentration of 100 μg/ml. CaCO_3_ particles were homogeneously dispersed in the composite solution, and as soon as GDL was added, CaCO_3_ started to dissolve and release Ca^2+^ ions to induce the crosslinking of the hydrogel. The gelling solution was continuously stirred for different times depending on the composition of the composite solution; then, each composite solution was pipetted into a 12-mm-diameter cylindrical mold. The gels were left at room temperature for 72 h to allow for complete gelation.

### Characterization

Alg-PDRN with a 1.2-mm diameter and 0.5-mm height was characterized using optical images to observe the transparency change and scanning electron microscopy (SEM; JSM-6360, JEOL, Japan) to observe their cross-sectional morphologies. The pore size of the hydrogels was measured using ImageJ software (National Institute of Health, Maryland, USA).

For the PDRN imaging, a silicon wafer was used as the substrate. The silicon substrate was cleansed with ethanol prior to the experiment. Because DNA molecules are negatively charged, the surface was positively glow discharged (PELCO easiGlow; TED PELLA Inc., USA) at 15 mA for 30 s. A monolayer of the concentrate was formed by uniformly distributing 10 µl of the PDRN solution (100 µg/ml, diluted in deionized water) on the silicon substrate. After 1 min, excess solution was blotted using filter paper. A topography image was obtained using AFM (NX-10; Park Systems, South Korea) with a non-contact tip.

The water uptake property of the hydrogels was evaluated by calculating the equilibrium swelling ratio. The hydrogels were first immersed in phosphate buffered saline (PBS) at 37 °C for 24 h; then, the weights of the swollen hydrogels were measured. The swelling ratio was calculated using the following equation:

$$\mathrm{Swelling\,ratio }\left(g/g\right)=\frac{W_{wet}-W_{dry}}{W_{dry}}$$

where W_wet_ is the weight of the swollen hydrogel and W_dry_ is the dry weight.

To determine the gelation time, 1 ml of the composite solution of each concentration was reacted under continuous stirring with a predetermined amount of GDL in a 10-ml vial. The vials were continuously tilted and inverted until the solution did not flow. The time required to achieve this condition was recorded as the gelation time.

Fourier-transform infrared (FT-IR) spectroscopy (Nicolet iS50, Thermo Fisher Scientific, USA) was used to verify the interaction between Alg and PDRN. FT-IR spectra of PDRN alone, Alg, and Alg-PDRN were obtained between 500 and 4000 cm^−1^.

### Rheological behavior

The rheological behaviors of the Alg and Alg-PDRN hydrogels were examined using a rheometer (ARES, Rheometric Scientific, USA)^52,53^. First, 1 ml of the hydrogels was placed between 20-mm-diameter parallel plates, and then, frequency sweep tests were performed under a constant strain of 0.5% in the frequency range of 1.0–100 rad/s. The storage modulus (G′) and loss modulus (G″) were measured as a function of frequency. The storage modulus of the hydrogel with and without PDRN at 1 Hz was also recorded for comparison.

### PDRN release behavior

The PDRN release behavior from the hydrogel carrier was quantified at 260 nm using UV–vis spectroscopy (NanoDrop; ThermoFisher Scientific, USA). To mimic the wound bed, a Franz-diffusion-cell-inspired apparatus was set up, as shown in Fig. S1. The Alg-PDRN was immersed in PBS for 30 min prior to the experiment to minimize the burst release effect and to concentrate PDRN at the center of the carrier, as the surface extraction technique is often used to control the initial burst release of a drug in drug delivery using hydrogel carriers^54^. A total of 3.4 ml of PBS solution was in contact with the lower surface of the hydrogels, being separated by a porous membrane composed of polyethylene and polypropylene (DASHI Bag; T&CE Electronics, South Korea). The release test was followed for 14 days, and the solution was tested at every time point for PDRN quantification. The solutions were filtered using a 0.2-µm syringe filter (syringe-driven filter; JET BIOFIL, China) to remove any impurities. The filtrates were then quantified by measuring the absorbance. The Alg and Alg-PDRN hydrogels were subjected to the same procedure, and the filtrates were used as the blanks.

### In-vitro cell proliferation, migration, and enzyme-linked immunosorbent assays

Cell proliferation and migration tests were performed using HDFs (PCS-201-012, ATCC, USA) and human diabetes mellitus (DM) fibroblasts which were obtained from diabetic foot patients from the department of plastic and reconstructive surgery of Seoul National University Boramae Hospital (IRB No. 26-2016-180). Cell proliferation tests using HUVECs (CRL-1730ATCC, USA) were also performed.

For the cell proliferation tests, the cells were seeded on the tissue culture plates with a density of 0.5 × 10^4^ cells/well, and the hydrogel samples were placed on top of the membrane with the cell culture medium in contact with the lower surface of the hydrogel for PDRN release. The cells were cultured in a humidified incubator in an air atmosphere containing 5% CO_2_ at 37 °C for 3 days. The cell culture medium was changed 3 times within 24 h to mimic the circulation system in real physiological conditions. The degree of cell viability was evaluated using a methoxyphenyl tetrazolium salt (MTS) assay (CellTiter 96 Aqueous One Solution, Promega, USA)^24^. Before the measurements, the cells were washed with PBS to remove non-adherent cells and immersed in minimum essential medium alpha containing 10% MTS and then reacted for 2 h. The quantity of formazan produced by the cellular activity was monitored by measuring the optical absorbance at 492-nm wavelength using a microplate reader.

To determine the effect of the released PDRN on fibroblast migration, an in vitro scratch assay was performed, as previously described in the literature^55^. A total of 1 × 10^6^ cells/ml were seeded on each side of the culture insert (Culture-Insert 2 Well in µ-Dish 35 mm, Ibidi, Germany) and cultured for 24 h. Then, the culture insert was removed to create a gap between the cell layers. After washing once with fresh cell culture medium, the cells were cultured for 10 h; µ-Dishes were placed inside a 6-well plate, and the hydrogels were placed on top of the membrane with the cell culture medium added until it was in contact with the lower surface of the hydrogel for PDRN release. The well without the hydrogel was used as the control group. After 10 h of culturing, optical images of each well were taken using an optical microscope (Oxion Inverso, Euromex, Holland). The proportions of the initial scratch (A_o_) and healing scratch (A_t_) were calculated using Image J software. The migration ratio (A) was determined using the following equation:

$$\mathrm{Migration\,ratio,\,A}\,({\%})=\frac{\mathrm{Ao}-\mathrm{At}}{\mathrm{Ao}}\times 100.$$

The cell culture supernatant was collected after 3 days of incubation with Alg-PDRN using the same method used for the proliferation test. The concentrations of cytokine were measured using enzyme-linked immunosorbent assay kits for VEGF and FGF (Invitrogen, Thermo Fisher Scientific, USA)^56^.

### In-ovo CAM assay

Fertilized chicken eggs were incubated at 37 °C in a humidified rotary incubator. At day 5 of embryonic development, windows were made on the egg shell for experiment. Alg and Alg-PDRN were placed on the chorioallantoic membrane, and the embryos were maintained for an additional 24 h at 37 °C in a stationary humidified incubator. CAM images were taken at 6 and 24 h. The images were then analyzed using Wimasis Image Analysis automatic software to quantify the angiogenesis. Data on the vessel density, total vessel network length, total branching points, and total segments were recorded.

### In-vivo assessment

An animal study was approved by the Institutional Animal Care and Use Committee (IACUC) of the Seoul National University Boramae Hospital (IACUC number 2019-0033). C57BLKS/J-db/db male mice (8 weeks, body weight 33.48 ± 3.13 g, plasma glucose 560.38 ± 72.19 mg/dL) were purchased from Sankyo Labo Service Corporation (Shizuoka, Japan). All the animal experiments were performed according to Seoul National University Hospital Institutional Animal Care and Use Committee guidelines.

All the mice underwent hair removal from the dorsal surface and sterilization of the skin with povidone-iodine and alcohol. In this wound model, four full-thickness wounds were created on each side of the midline using a sterile 8-mm punch biopsy tool (Miltex, Inc. PA, USA). Silicone splints were sutured around the defect^57^. Saline and PDRN were subcutaneously injected at five sites after the wound was created. Then, Alg and Alg-PDRN were placed on the wound. After the surgery, the wounds were covered with Opsite Flexifix (Smith and Nephew, Watford, UK), and the wounds were wrapped with a Peha-haft bandage (Hartmann, Heidenheim, Germany). After the surgery, the mice were carried in individual cages (n = 5). To observe the effect of saline and PDRN injection and Alg and Alg-PDRN treatments on the wound area, the wounds in db/db mice were monitored using a digital camera. Digital photographs were taken at days 0, 1, 3, 6, 8, 10, and 14, and the wound area was assessed using National Institutes of Health Image J 1.36b imaging software (National Institutes of Health, Bethesda, USA).

The wounds were harvested at day 14 after surgery and fixed in 4% paraformaldehyde for 24 h at 4 °C, washed with water for at least 4 h, and embedded in paraffin. The paraffin-embedded sections of 4-µm-thick samples were stained with hematoxylin and eosin (H&E, ab245880, Abcam, Cambridge, UK) and Masson’s trichrome (M-T, TRM-2, ScyTek, Utah, USA) stains according to the manufacturer’s guidelines. The cellularity of the H&E stained image at 400 × magnification and the collagen density of the M-T stained image at 100 × magnification were determined using an Olympus BX53 microscope (Olympus Corporation, Shinjuku, Japan) and captured from three microscopic fields (right, center, and left). The number of cells per unit area was calculated using the Olympus cellSens standard imaging software. The optical density of the collagen density was measured using National Institutes of Health Image J 1.36b imaging software. Immunohistochemical (IHC) analysis was performed on 4-µm paraffin sections of the wound skin samples. Briefly, skin sections were de-paraffin processed, and tissue sections were blocked with tris buffered saline (TBS) containing 1.5% normal bovine serum for 1 h at room temperature. The sections were then incubated with mouse monoclonal transforming growth factor-beta 1 (TGF-β, 1:100), rabbit polyclonal myeloperoxidase (MPO, 1:300), rabbit polyclonal alpha-smooth muscle actin (α-SMA, 1:2000), and mouse monoclonal vascular endothelial growth factor (VEGF, 1:300) in blocking solution overnight at 4 °C. After washing two times in TBS containing 0.025% Triton X-100 (TBS-T), the sections were incubated in secondary antibodies for 30 min at room temperature. Images (right, center and left) were captured using an Olympus BX53 microscope. The optical density of TGF-β, MPO, α-SMA, and VEGF expression and collagen density were measured using National Institutes of Health Image J 1.36b imaging software.

The following primary antibodies were used for the IHC analysis: transforming growth factor-beta 1 (TGF-β, MA240; R&D, Minneapolis, USA), myeloperoxidase (MPO, A0398; Dako, Glostrup, Denmark), alpha-smooth muscle actin (α-SMA, 14395-1-AP; Proteintech, IL, USA), and vascular endothelial growth factor (VEGF, sc-57496; Santa Cruz, Texas, USA). The following secondary antibodies were used for IHC analysis: peroxidase rabbit IgG (PK-4001; Vector Laboratories, Burlingame, CA) and peroxidase mouse IgG (PK-4002; Vector Laboratories, Burlingame, CA).

### Statistical analysis

Experiments were performed with sample sizes greater than three, and the experimental results are expressed as the mean value ± standard deviation. The difference between the groups was determined using one-way analysis of variance, and a *p* value smaller than 0.05 was considered a statistically significant difference.

For the in vivo experiment, all the values are reported as the mean ± standard error of the mean (SEM). Statistical tests were performed using GraphPad Prism 7.0 software. Statistical significance was determined using the Mann–Whitney U nonparametric test, and multiple comparisons were tested with two-way ANOVA. A *p* value of < 0.05 was considered statistically significant.

## Supplementary information

Supplementary file1
