# Supplementary material for: Polydeoxyribonucleotide-delivering therapeutic hydrogel for diabetic wound healing
Source: Sci Rep. 2020 Oct 8;10:16811. doi: 10.1038/s41598-020-74004-0 (PMC7546631; doi:10.1038/s41598-020-74004-0)
Supplement: Supplementary file 1 — Supplementary file1 [file 41598_2020_74004_MOESM1_ESM.docx]

**Polydeoxyribonucleotide-Delivering Therapeutic Hydrogel for Diabetic Wound Healing**

Da Yong Shin^a, ⊥^, Ji-Ung Park^b, ⊥^, Min-Ha Choi^b^, Sukwha Kim^c^, Hyoun-Ee Kim^a,d^, Seol-Ha Jeong^a,^*

a Department of Materials Science and Engineering, Seoul National University, Seoul, 08826, Republic of Korea

b Department of Plastic and Reconstructive Surgery, Seoul National University Boramae Medical Center, Seoul, 07061, Korea

c Medical Big Data Research Center, Seoul National University College

of Medicine, Seoul, 03080, Korea

d Advanced Institutes of Convergence Technology, Seoul National University, Gwanggyo, Yeongtong-gu, Suwon-si, Gyeonggi-do, 16229, Republic of Korea

* Corresponding author

E-mail address: jsh528@snu.ac.kr

**Table S1.**

Experimental conditions for alginate hydrogel with varying concentration.

| **Sample** | **Alg Conc. [w/v%]** | **CaCO_3_ Conc. [mM]** | **GDL Conc. [mM]** |
| --- | --- | --- | --- |
| 2% Alg | 2 | 25 | 50 |
| 3% Alg | 3 | 37.5 | 75 |
| 4% Alg | 4 | 50 | 100 |
| 5% Alg | 5 | 62.5 | 125 |


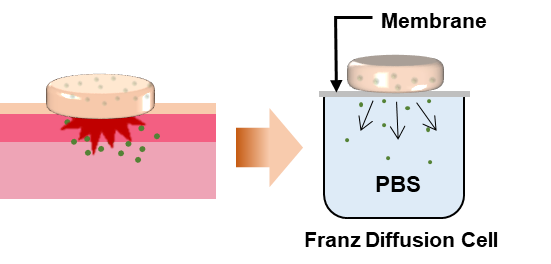


**Figure S1.** Schematic illustration of the Franz diffusion cell inspired apparatus that mimics the real application of the hydrogel sheet (porous membrane separating the hydrogel and PBS medium at the bottom for the collection of the released PDRN).

**Figure S2.** PDRN absorbance spectra at 260 nm with varying concentration and absorbance of PDRN diffused from the hydrogel.


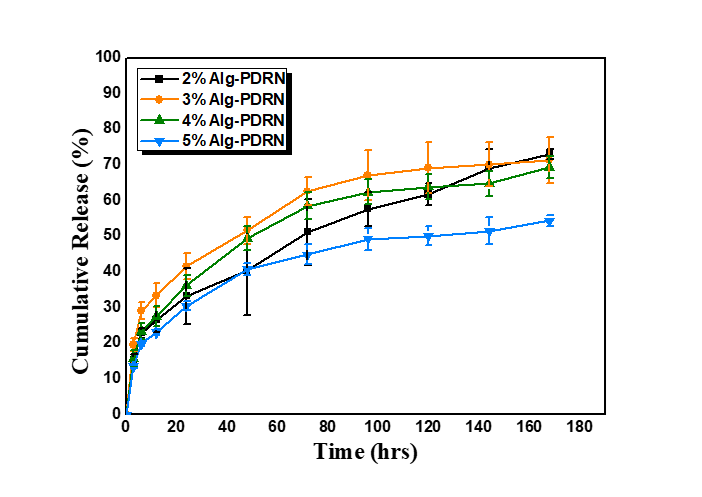


**Figure S3.** The releasing profile of the Alg-PDRN hydrogels with different alginate concentration in cumulative percent release.


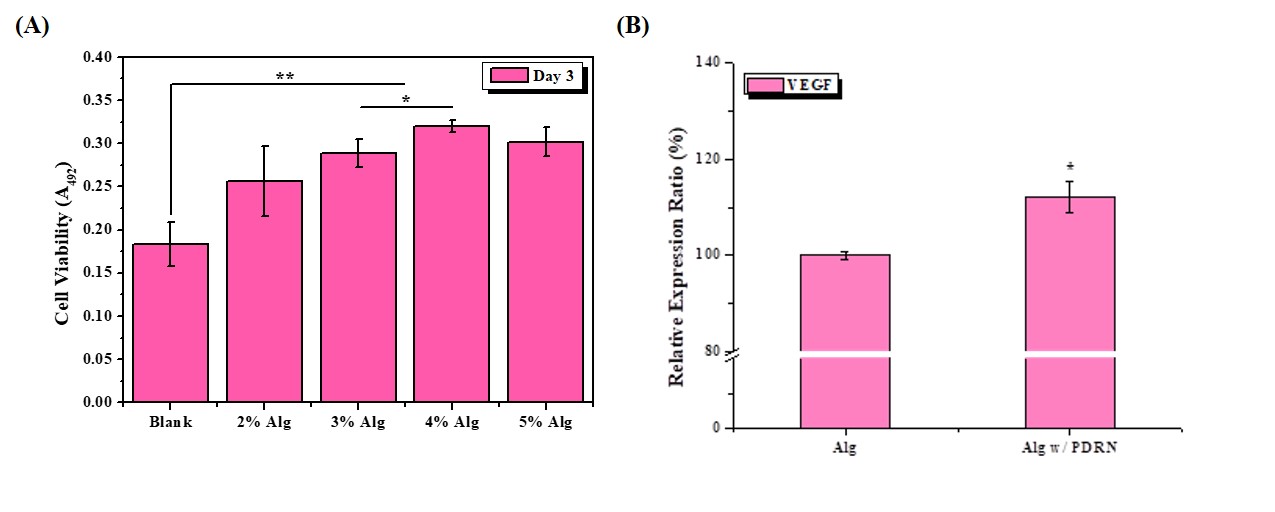


**Figure S4.** (A) Cell viability of HUVEC treated with PDRN loaded alginate hydrogels for 3 days and (B) VEGF expression from HUVEC treated with Alg and Alg w/ PDRN (* p < 0.05, ** p<0.005)
